# The Relations of Dental Diseases to General Diseases

**Published:** 1899-08

**Authors:** William Hunter


### Abstracts and Translations.

### THE RELATIONS OF DENTAL DISEASES TO GENERAL DISEASES.

BY WILLIAM HUNTER, M.D., F.R.C.P.

It is now some ten years since, in the course of a somewhat detailed study of the pathology of the severest forms of anæmia, my attention was drawn to the mouth as a possible source of the infection which, according to my observations, undoubtedly underlies these forms of anæmia. . . .

To-night I propose, therefore, to confine my remarks within certain defined limits, to pass over briefly those diseases whose relation to dental disease is obvious and well recognized, and to direct attention to those—in my opinion, no less important—where this relation is not so clear, yet probably hardly less close. . . .

### BACTERIOLOGY OF DENTAL CARIES.

On this point—the infective nature of dental caries—I need not dwell. The evidence so abundantly furnished by the laborious bacteriological observations of Miller (1884–1894), on no fewer than two hundred and fifty cases of diseased teeth; Galippe and Vignal (1889), Jung (1893), and most recently of all by Professor Arkövy, of Budapesth (1898), is to my mind overwhelmingly conclusive on this point.

The observations of the last mentioned seem to me to be of such particular importance that I venture to summarize them very briefly. They extend from 1878.

He has studied in detail the organisms found in the following conditions:

1. Chronic alveolar abscess, with and without parulis.
2. Gangrene of pulp, both acute and chronic.
3. Old stoppings removed after varying periods of time.
4. Chronic alveolar abscess with circumscribed alveolar necrosis, after previous root-stopping.

His method was as follows:

After complete evacuation of the pus from the abscess-cavity, or removal of the gangrenous pulp from the pulp-cavity, and out of the root as far as the apical foramen, all parts were thoroughly disinfected, first, with one per cent. corrosive sublimate, and then with pure carbolic acid, and then thoroughly packed with a jelly-like mixture of camphor, pure carbolic, and oil of eucalyptus, and the whole closed in with a covering of asbestos and gutta-percha. In this condition the tooth was left some three to six months before the definitive filling was proceeded with.

Many cases under this treatment healed entirely, and he regarded them as sterile. A small minority still presented some degree of parulis or chronic periostitis remaining; and the cause of this he endeavored to ascertain by careful cultivation, the pathogenic properties of the various cultures being determined by experiments on animals.

The number of cases examined in this way was forty-three; and the chief result of his observations is to show that the organism most constantly present in diseased pulps and in dental caries is a bacillus to which he gives the name of *bacillus gangrænæ pulpæ.* Its relative frequency, as compared with other organisms, is 95.3 per cent. of all cases.

Next most frequent is the staphylococcus pyogenes aureus in 34.8 per cent. of cases; then the streptococcus pyogenes in 23.2 per cent. of cases; then staphylococcus pyogenes albus, 18.6 per cent.; staphylococcus pyogenes citreus, 4.6 per cent.; bacillus pyocyaneus, 9.3 per cent., and some nine other organisms, mostly harmless, in varying frequency.

Morphologically the characteristic feature of bacillus gangrænæ pulpæ is that it is pleomorphic, forming bacilli when grown on gelatin, cocci when grown on agar-agar.

As regards pathogenic action, pure cultures of this organism were found to possess the power, single-handed, of producing gangrene of the pulp; never suppuration, unless other organisms were present.

A further important observation made was, this organism could effect a softening of the tooth, even in an alkaline medium, a fact which, if confirmed, would dispose of the view widely prevalent regarding dental caries,—viz., that the first decalcification is the result of the action of an acid.

The organisms found in carious dentine after sterilization were: bacillus gangrænæ pulpæ in every one; staphylococcus pyogenes aureus; streptococcus pyogenes; bacillus gangrænæ pulpæ the only constant.

The pyogenic organisms were always absent in teeth properly dealt with antiseptically.

### RELATION TO LOCAL INFECTIVE DISEASES.

This group includes not only the various complications of diseased teeth met with in the bones of the jaw, the gums, neighboring maxillary sinuses,—*e.g.*, alveolar abscess, suppuration in the sockets (pyorrhœa alveolaris), periostitis, osteitis, osteomyelitis, necrosis of bone, suppuration in maxillary sinuses,—but also inflammations and suppuration in neighboring parts by direct extension, such as inflammation, and at times suppuration of lymph-glands of neck, cellulitis of neck, post-pharyngeal abscess, thrombosis of veins, meningitis.

This group of cases may, perhaps with one exception, be excluded from our field of survey to-night. Their relation to dental disease is obvious, and does not require any special elucidation.

Cases of this kind are not uncommon. A number are on record (one such came but recently under my notice) where, after extraction of a tooth with a foul instrument, a condition of gangrenous stomatitis, osteomyelitis, and necrosis has been set up, and death has ensued from pyæmia.

### DISEASED GLANDS IN NECK.

One of the conditions above referred to deserves a more detailed notice,—viz., the relation of dental caries to chronic glandular enlargements in the neck. To what extent may such enlargements be due in the first instance to conditions of decay in the teeth,—to irritation set up by inflammatory conditions around the teeth in children; the chronically enlarged glands subsequently forming favorable seats of infection for tubercle bacilli, with all the troubles attendant on tuberculous glands of neck.

Odenthal [1] examined 987 children and found decayed teeth in 429. In 558 no decay.

Of the 558 without decayed teeth: glandular swellings in 275 = forty-nine per cent. Of the 429 with decayed teeth: glandular swellings in 424 = ninety-nine per cent.

He was able to determine a constant relation betwixt the extent of the glandular swellings and the extent of the decay.

---

[1] Odenthal, 1887, Caries of Teeth as Centres of Infection and Cause of Chronic Glandular Swellings of the Neck.

Wherever the pulp was gangrenous or highly inflamed the glandular swelling was invariably more pronounced and extended.

The presence of a number of decayed teeth was always accompanied by very marked glandular swellings.

The most recent contribution to the subject is that of Hugo Starck, "Tuberculous Cervical Glands in Relation to Carious Teeth" (*Münchener medicinische Wochenschrift,* vol. xliii., 1896).

He examined one hundred and thirteen children with glandular swellings of neck. In forty-one per cent. he found carious teeth, and in almost all of these the situation of the glands corresponded to the affected teeth.

In three children with tuberculous glands of neck he also found carious teeth; but he could not discover any tubercle bacilli in these.

In a girl, aged fourteen, otherwise healthy, he found a molar tooth containing tuberculous granulation tissue, and tuberculous glands on this side, and this alone.

This case suggests the possibility of a causal connection betwixt carious teeth and tuberculous glandular enlargements, but at the same time suggests that a local tuberculous lesion of the tooth is necessary, and this is very rare.

So far as the mouth is concerned, by far the most important seat of infection for tubercle is the tonsil.

Not only are giant-cells to be found in the tonsils in a considerable proportion of cases,—seven per cent. according to Cornil (*La Semaine médicale,* 1895, p. 223, seventy observations); but experiments have shown that tonsillar tissue, when injected into animals, is capable of giving rise to tuberculosis in a considerable percentage of cases,—according to Dieulafoy, thirteen per cent. (*La Semaine médicale,* 1895, p. 199, sixty-one experiments on guinea-pigs).

Not only for tubercle bacilli, but even more for pyogenic organisms, does the tonsil play an important part as a seat of entrance.

According to Buscke ("The Tonsils as Portals of Entry for Pyogenic Organisms," *Deutsche Zeitschrift für Chirurgie,* 1894), the tonsils play a greater *rôle* in admitting pus organisms than even the skin or the mucous membranes.

He found staphylococci and streptococci in hypertrophied tonsils which were not seats of acute inflammatory change.

He gives the history of four obscure cases of suppuration, in which the tonsils were the probable seat of infection, one of them a case of uncomplicated simple fracture, which pursued a normal course for three weeks, by which time a large amount of callus was thrown out.

On the twenty-sixth day the patient fell ill with a sore throat, due to streptococci. Three days later similar streptococci could be found in the blood, and on the following day the patient was seized with rigors and rapidly developed a suppurative osteomyelitis and periostitis at the seat of fracture.

(To be continued.)

